# Supplementary material for: Mechanisms governing the pioneering and redistribution capabilities of the non-classical pioneer PU.1
Source: Nat Commun. 2020 Jan 21;11:402. doi: 10.1038/s41467-019-13960-2 (PMC6972792; doi:10.1038/s41467-019-13960-2)
Supplement: Supplementary file 7 — Source data [file 41467_2019_13960_MOESM7_ESM.zip › Source_Data/FigureS2/FigureS2B_MotifScanOutput/KmeansCluster9/homerResults/motif17.similar.html]

motif17

## Information for motif17

A
C
T
G
A
T
G
C
C
G
T
A
A
C
T
G
A
C
G
T
A
C
G
T
A
G
T
C
A
G
T
C
A
G
T
C
A
G
C
T
T
G
C
A
T
C
G
A
G
C
T
A
  
Reverse Opposite:  

C
G
A
T
A
C
G
T
A
C
G
T
T
C
G
A
T
C
A
G
C
T
A
G
C
T
A
G
C
G
T
A
C
G
T
A
G
T
A
C
C
G
A
T
A
T
C
G
T
G
A
C
  

|  |  |
| --- | --- |
| p-value: | 1e-24 |
| log p-value: | -5.715e+01 |
| Information Content per bp: | 1.727 |
| Number of Target Sequences with motif | 65.0 |
| Percentage of Target Sequences with motif | 2.86% |
| Number of Background Sequences with motif | 232.7 |
| Percentage of Background Sequences with motif | 0.50% |
| Average Position of motif in Targets | 98.9 +/- 39.5bp |
| Average Position of motif in Background | 90.4 +/- 60.8bp |
| Strand Bias (log2 ratio + to - strand density) | -0.2 |
| Multiplicity (# of sites on avg that occur together) | 1.00 |
| Motif File: | file (matrix) reverse opposite |

### Similar de novo motifs found

|  |  |  |  |  |  |  |  |
| --- | --- | --- | --- | --- | --- | --- | --- |
| Rank | Match Score | Redundant Motif | P-value | log P-value | % of Targets | % of Background | Motif file |
| 1 | 0.752 | G T C A C G A T T G C A G T A C C T A G T A C G G T C A C T G A T G A C G A C T A C T G | 1e-24 | -56.987382 | 5.76% | 1.92% | motif file (matrix) |
| 2 | 0.850 | C G A T C T A G C T A G C T A G C T A G C T G A T C G A T G C A C G A T C A T G T A G C G T C A | 1e-13 | -31.557356 | 1.27% | 0.18% | motif file (matrix) |
